# The immune response to *Francisella tularensis*

**DOI:** 10.3389/fmicb.2025.1549343

**Published:** 2025-04-25

**Authors:** Derek A. Barthels, Robert V. House, H. Carl Gelhaus

**Affiliations:** ^1^Department of Biology, Life Sciences Research Center, United States Air Force Academy, Colorado Springs, CO, United States; ^2^National Research Council Research Associateships Program, Washington, DC, United States; ^3^Dr. RV House LLC, Harpers Ferry, WV, United States; ^4^Appili Therapeutics, Halifax, NS, Canada

**Keywords:** *Francisella tularensis*, tularemia, adaptive immunity, innate immunity, correlates of protection, vaccine

## Abstract

*Francisella tularensis* (*Ft*) is a Gram negative intracellular bacterial pathogen, commonly transmitted *via* arthropod bites, but is most lethal when contracted *via* inhalation. The nature of a Gram-negative intracellular pathogen presents unique challenges to the mammalian immune response, unlike more common viral pathogens and extracellular bacterial pathogens. The current literature on *Ft* involves numerous variables, including the use of differing research strains and variation in animal models. This review aims to consolidate much of the recent literature on *Ft* to suggest promising research to better understand the complex immune response to this bacterium.

## 1 Introduction

*Francisella tularensis* (*Ft*) is a highly infectious Gram-negative, facultative intracellular coccobacillus; infection with *Ft* results in the disease tularemia. Clinical tularemia manifestations depend on the site of infection, with ulceroglandular, glandular, oculoglandular, oropharyngeal, pneumonic, and typhoidal forms described (Cummings et al., 2014). Whereas the ulceroglandular form of the disease is the most common naturally acquired form (usually *via* arthropod bites), the pneumonic form (infection *via* inhalation) exhibits the most serious pathology and has one of the highest mortality rates among all tularemia forms. Fortunately, naturally occurring *Ft* infections are rare, with approximately 200 infections reported annually in the US (Centers for Disease Control and Prevention, 2022).

The genus *Francisella* has over a dozen species, with *Ft* being the mammalian pathogenic species (Degabriel et al., 2023). Human pathogenic *Ft* is further subdivided into three subspecies by virulence and geographic distribution: *Ft tularensis* (*Ftt*, most virulent, found in North America), *Ft holartica* (*Fth*, moderate virulence, found throughout the northern hemisphere), and *Ft mediasiatica* (*Ftm*, lowest virulence, found in central Asia) (Larson et al., 2020). *Ft* has long been considered an important biological warfare/bioterrorism threat and has been weaponized by both the US and the former Soviet Union (McLendon et al., 2006; Janik et al., 2020). Characteristics of *Ft* that support its use as a bioweapon include a very low infectious dose, ease of aerosolization, multiple debilitating and potentially fatal clinical syndromes, suboptimal antibiotic countermeasures requiring prolonged therapy, and lack of effective vaccines. In addition, tularemia is an important zoonotic disease that is transmitted by various arthropod vectors (Yeni et al., 2021).

Efforts to develop and license an effective tularemia vaccine have been underway since the 1940s (Griffin et al., 2007). Efforts have included live attenuated vaccines for tularemia, with the preponderance of data using the Live Vaccine Strain (LVS) candidate. The LVS vaccine candidate was generated from an *Fth* strain in the USSR and tested in human volunteers who were vaccinated and then challenged with virulent *Ft via* intracutaneous (Saslaw et al., 1961) and respiratory (Saslaw et al., 1961) challenge routes. United States (US) published studies were conducted at the Ohio State Penitentiary on inmates who agreed to participate in the clinical trial. Additional studies were conducted by the US military at Fort Detrick under Operation Whitecoat, where researchers conducted 40 clinical studies of tularemia from 1958 to 1968 (Pittman et al., 2005). These studies used male volunteers on active duty in the Army, including members of the Seventh Day Adventist Church who were conscientious objectors. In these studies, the volunteers were exposed to the SCHU S4 strain of *Ft*. LVS was shown to protect against aerosol challenge, albeit at low challenge doses (Saslaw et al., 1961). There are extensive data on the history of the immune response to LVS.

LVS has many advantages in the study of *Ft*, primarily because of its reduced virulence; it is safer for researchers to use and is exempt from select agent regulations. However, as a less virulent subspecies, it may not elicit the same immune response as the more virulent *Ftt* subspecies. Recently, more laboratories have been using the SCHU S4 strain of *Ftt*, and there is increasing immunological literature using *Ftt*. In addition, a SCHU S4 based live attenuated tularemia vaccine is reported to be in development.

It should be noted that extensive literature exists regarding the role of T cells in the immune response to *Ft* and particularly to a second infection or infection following challenge with a live attenuated strain. Early studies on the need for T cell immunity to protect against a second *Ft* exposure have shown that BALB/c mice infected intradermally with LVS died, whereas BALB/c nu/nu mice (which lack a thymus and T cells) lived (Elkins et al., 1993). Furthermore, the addition of either CD4^+^ or CD8^+^ T cells to BALB/c scid (lymphocyte deficient) mice restored the protection from intradermal LVS infection (Elkins et al., 1996). Similar results were observed with antibody depletion of CD4^+^ and CD8^+^ T cells in BALB/c mice infected with LVS (Yee et al., 1996). The extensive role of ɑꞵ T cells in tularemia vaccines has been reviewed recently by others (Roberts et al., 2018; Harrell et al., 2024) and is not discussed here. This is not intended to diminish the role of T cells in *Ft* infections, as it is substantial, but rather to recognize that this topic is well addressed by other recent reviews.

Another historical aspect of the immune response to *Ft* is the involvement of B cells and the production of antibodies. It is well known that antibodies are produced in response to Ft infection and these antibodies are used to determine the seroprevalence of *Ft* in many environmental species. Human antibody production after *Ft* exposure has been used as a diagnostic tool as well as a tool to measure immune response to LVS vaccination (Maurin, 2020; Koskela, 1985). However, antibodies have been shown to give protection only against strains of reduced virulence (Tarnvik, 1989). The role of antibodies and B cells has been recently reviewed by others (Kubelkova and Macela, 2021) and is not discussed here. As with T cells, this is not to diminish the role of antibodies or B cells, but to focus this review on other parts of the immune response.

**Figure 1:**
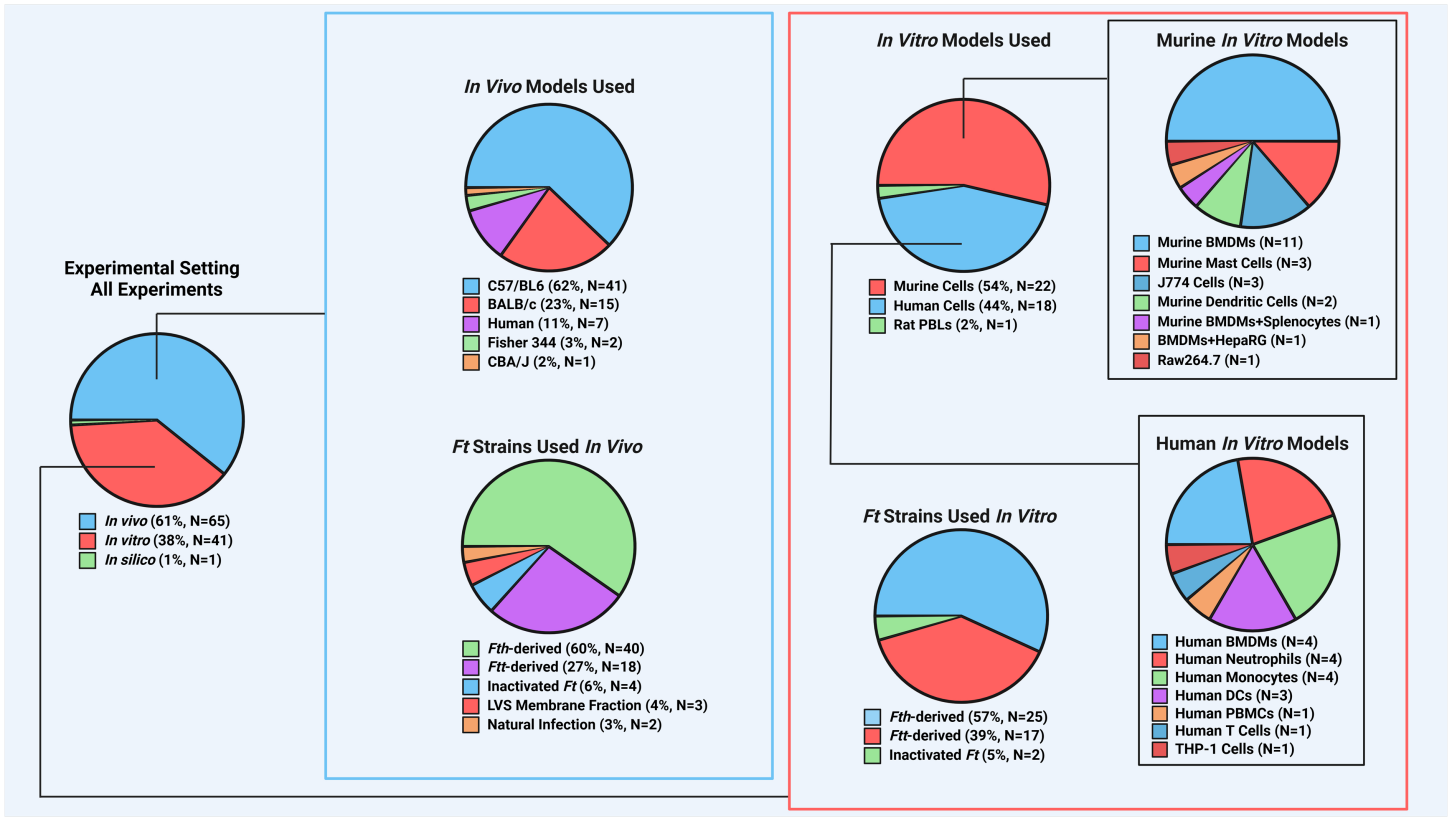
Breakdown of experimental models found in recent *Ft* literature. To better understand the distribution of experimental models in studies, counts were recorded for each model aspect, including species used for *in vivo* and *in vitro* experiments and the frequency of specific *Ft* strains. For instance, if a publication used *Fth*, *Ftt*, BALB/c mice, and C57BL/6 mice, each was counted once. Mouse models dominated *in vivo* studies (~86%), with *Fth*-derived strains being the most common. This highlights a need for more research on other animal models. *In vitro* studies were more balanced, with mouse models comprising ~54% and a more even use of *Fth* and *Ftt* strains. (*Ft*: *Francisella tularensis*, *Fth*: *Francisella tularensis* subsp. *holarctica*, *Ftt*: *Francisella tularensis* subsp. *tularensis*, LVS: live vaccine strain, PBL: peripheral blood leukocyte, BMDM: bone marrow-derived macrophage, PBMC: peripheral blood mononuclear cell).

While certain aspects of the immune response to *Ft* infection have been well established, we review other aspects of the immune response to *Ft*, including molecular responses, innate immune responses and some less-considered aspects of the adaptive immune response (Table 1). Differences in the response to *Fth* vs. *Ftt* are described, including differences in toll-like receptors (TLRs), inhibition of reactive oxygen species (ROS), interferon (IFN)-*γ* and other cytokine responses, cellular depletion, cellular expansion responses, and tissue necrosis. We review the literature to gain more detailed understanding of the immune response to *Ft* and the differences in responses among the subspecies. The complete immune response, considering all cellular and molecular aspects, has not been fully established. This review summarizes the state of knowledge based on the past decade of research and identifies areas for future research. We examine a variety of experimental models recently used (Figure 1). We conclude that *Ft* is an inherently immunomodulatory organism, but that differences in animal models, including routes of infection and/or immunization, have resulted in conflicting results. Furthermore, inconsistencies in the *Ft* subspecies and strain used cause differential activation of various molecular pathways, leading to divergent conclusions from the respective data. Though caveats in the models and bacterial strains are important and should be acknowledged, there remains an abundance of evidence that offers valuable insights into the pathophysiology and mechanisms of *Ft* infection.

**Table 1:** Comparison of experimental models used in *Ft* literature.

Pathogen recognition and response mechanisms	Different response to Ftt or Fth?	Different response based on experimental setting?
Inflammasomes	No direct comparisons	Bacterial growth conditions may affect *in vitro* results
Toll-like receptors	TLR4 detects *Fth* but not *Ftt*	No comparison across species or models
Suppression of ROS	*Ftt* more robustly inhibits ROS production *in vitro*	No comparison across species or models

Innate immune cells	Different response to *Ftt* or *Fth*?	Different response based on experimental setting?
Macrophages	Stronger IFN-γ and NO responses from attenuated *Ftt*	No comparison across species or models
Dendritic cells	*Fth* induced more robust cytokine production *in vitro*	No comparison across species or models
NK cells	*Ftt* depletes NK and T cells by 90% *in vivo*, induced different IFN-γ response timeline	No comparison across species or models
Mast cells	No key differences between strains	No comparison across species or models
Neutrophils	Lower dose of *Ftt* causes neutrophil-induced lung necrosis after inhalational exposure	No comparison across species or models
γδ T cells	*Fth* induces slower expansion of *γ*δ T cells	Vγ9/Vδ2 T cell subset is specific to primates
iNKT cells	No direct comparisons	No comparison across species or models
MAIT cells	*Ftt* not used in any study identified in literature search	No comparison across species or models

Adaptive immune cells	Different response to *Ftt* or *Fth*?	Different response based on experimental setting?
B cells	*Ftt* depletes NK and T cells by 90% *in vivo*, induced different IFN-γ response timeline	No comparison across species or models
T cells	No direct comparisons	No comparison across species or models

While it is difficult to summarize all the differences seen between the wide variety of model combinations in the literature, some noticeable variations between models were found that could meaningfully alter experimental results. To account for the complexities of the models and avoid unsubstantiated assumptions, comparisons were made only when multiple *Ft* strains, experimental settings, and/or animal species were explicitly used for comparison (TLR: toll-like receptor, ROS: reactive oxygen species, NK cells: natural killer cells, iNKT cells: innate natural killer T cells, MAIT cells: mucosal-associated invariant T cells, *Ftt*: *Francisella tularensis* subsp. *tularensis*, *Fth*: *Francisella tularensis* supsp. *Holarctica*; IFN, interferon; NO, nitric oxide).

## 2 Methods

### Review Criteria

Our systematic review identified not only recent cellular immunity and cytokine-mediated immunity studies, but also molecular changes. First, the molecular changes are reviewed below.

The purpose of this literature-based critical review is to identify changes following *Ft* exposure; changes associated with the immune system with an interest in those changes that could be assessed to guide development and testing of MCM specific for tularemia. It is crucial to note that the search for useful correlates of protection for *Ft* is complicated by observations that the nature and course of infection with *Ft*, as well as mechanisms of protection, varies significantly depending on route of exposure (Nicol et al., 2021). This difference is important when designing an *Ft* MCM development program, since the indication (i.e., the expected route of exposure) will drive the testing design. More specifically, although naturally occurring tularemia results from arthropod bites (i.d. exposure), these infections are milder and often self-limiting. Respiratory exposure is rare in nature but would be the expected route of exposure if *Ft* were used as a bioweapon. In this case, the disease would be far more dangerous with an untreated mortality rate of approximately 60% (Hong et al., 2013). Here, we review the immunological literature against the backdrop of a tularemia MCM intended to prevent pneumonic tularemia following a respiratory exposure in humans.

The following strategy was used in the current review:

1. An initial search was conducted in PubMed using search terms” tular* innate” and “tular* adaptive.”
2. The Inclusion/Exclusion criteria and screening process included:
  1. Papers published in 2013 or later in scientific journals
  2. Relevant to *Ft* and its mechanisms of infection/evasion once inside the host
  3. Only articles in English were reviewed
  4. Initial search included only research papers (not reviews)
3. This initial search identified several hundred primary publications that met the search strategy.
4. The publications were collated in Qiqqa (https://bit.ly/45f6a3U, RRID:SCR_004440), an open-source citation management program that employs tags and metadata to organize the publications and allows collation of keywords and topics. Qiqqa identified several common terms including absent in melanoma (AIM)2, Cas9, CRISPR, Caspases, IRF3, macrophages, ROS, T6SS, and TLR2/4. Additional mechanisms were identified during review of the literature.
5. Information from additional resources (reviews and other relevant publications) outside of the studies identified by the initial search was subsequently included for context.

Thirty papers were reviewed and found to refer more to *Francisella* biology (e.g., virulence factors) than to host immune responses, including six papers on CRISPR/Cas. These papers were not included in the literature findings.

## 3 Results

### Literature findings

### 3.1 Molecular defense mechanism changes

Our systematic review identified not only recent cellular immunity and cytokine-mediated immunity studies, but also molecular changes. First, the molecular changes are reviewed below.

#### 3.1.1 Genetic mechanisms of *Ft* immune evasion

SCHU S4 and LVS use genetic methods to evade the immune system, such as leveraging host microRNAs (miRNAs) to inhibit messenger RNA (mRNA) translation. Notably, miR-155 broadly suppresses the host inflammatory response in macrophages, B cells, and T cells. This miRNA, along with miR-150 and miR-146, which target macrophage function, is upregulated in human bone marrow-derived macrophages (BMDMs) after infection with SCHU S4 and LVS. Bioinformatics analysis also identified SH2-containing inositol phosphatase-1 (SHIP-1) and myeloid differentiation primary response 88 (MyD88) as potential targets of miR-155 (Bandyopadhyay et al., 2014). The potential blocking of SHIP-1 by miR-155 is intriguing. SHIP-1 indirectly inhibits the Akt pathway by dephosphorylating phosphatidylinositol triphosphate (PIP3), which is crucial for Akt pathway activity. Thus, SHIP-1 hinders basic cellular functions like growth, proliferation, and survival (Cremer et al., 2011). If miR-155 is induced by infection in non-macrophages, it could enhance these processes, promoting cell survival.

**Figure 2:**
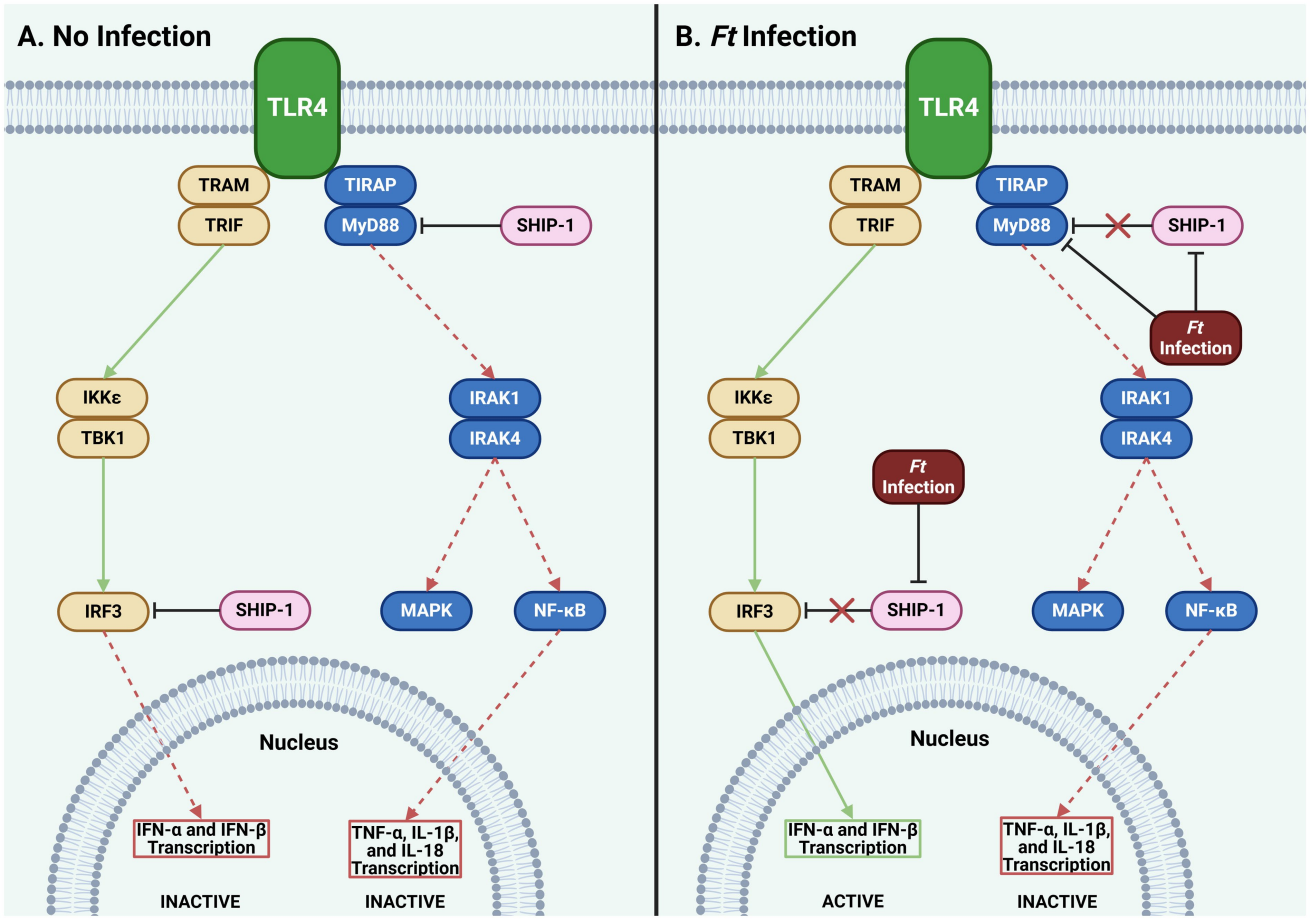
Effect of *Ft* infection on TLR4 signaling. **(A)** SHIP-1 inhibits MyD88 and IRF3, blocking transcription of type I IFNs, TNF-*α*, IL-1*β*, and IL-18. **(B)** *Ft* infection inhibits SHIP-1, typically engaging IRF3 and MyD88. Though a second, unidentified mechanism, *Ft* simultaneously continues to inhibit MyD88. This results in the transcription of type I IFN genes while the transcription of TNF- α, IL-1β, and IL-18 remains suppressed. While these mechanisms are specific to only a subset of cells, they may provide insights to help accelerate a more robust innate immune response to *Ft* (*Ft*: *Francisella tularensis*, IFN: interferon, IKK: IκB kinase, IL: interleukin, IRAK: interleukin receptor-associated kinase, IRF: interferon regulatory factor, MAPK: mitogen-activated protein kinase, MyD88: myeloid differentiation primary response 88, NF-κB: nuclear factor-κB, SHIP: Src homology 2 domain containing inositol polyphosphate 5-phosphatase, TBK: TANK-binding kinase, TIRAP: toll-interleukin-1 receptor domain-containing adaptor protein, TLR: toll-like receptor, TNF: tumor necrosis factor, TRAM: translocating chain-associating membrane protein, TRIF: TIR-domain-containing adapter-inducing interferon-β).

miR-155 targets MyD88, a key player in TLR signaling that triggers inflammatory cytokines like tumor necrosis factor (TNF)-*α* and interleukin (IL)-1*β*. SHIP-1 also participates in TLR signaling by inhibiting MyD88 and its counterpart, TIR domain-containing adapter molecule (TIRAM)/TIR domain-containing adapter-inducing INF-β (TRIF). Interestingly, miR-155 blocks both MyD88 and SHIP-1, disinhibiting the MyD88 and TIRAM pathways but ultimately shuts down the MyD88 branch, leaving only the TIRAM pathway active (Figure 2). Bandyopadhyay et al. (2014) noted that miR-155 expression increases over time post-infection, favoring the TIRAM pathway and leading to late-stage production of IFN-α and IFN-β in tularemia (Furuya et al., 2014). The full implications of these interactions in innate immune responses to tularemia remain unclear and need further exploration.

SHIP-1 is also active in adaptive immune cells like B cells, T cells, mast cells, and natural killer (NK) cells, generally reducing their signaling (Conde et al., 2011). Since these cells are not primary targets for *Ft* infection, it’s unclear if miR-155 would be upregulated in them through paracrine or other signaling methods. The literature has not explored miR-155 activity in these cells post-tularemia infection, making it a promising research area for understanding *Ft*’s immune evasion. Future studies should also consider the roles of other miRNAs upregulated after tularemia infection and whether SHIP-2 could compensate for the lack of SHIP-1 activity.

The literature on *Ft*’s genetic immune evasion is limited compared to studies on protein and cytokine interactions between host and pathogen cells. Future research may reveal important genetic interactions with *Ft* and its hosts, but for now, most efforts focus on understanding protein interactions during *Ft* infections.

#### 3.1.2 Inflammasome

Intracellular pathogens like *Ft* evade extracellular defenses by replicating inside host cells. Hosts counteract these threats by triggering regulated cell death pathways, such as apoptosis, pyroptosis, and necroptosis. Inflammasomes, protein complexes with sensor proteins, detect pathogen virulence traits directly or indirectly and cause proinflammatory cytokine release and cell death (Nozaki et al., 2022). Common inflammasome mechanisms involve cytoplasmic NOD-like receptors (NLR) and absent in melanoma (AIM) 2. NLR pathways, activated by pathogen patterns and secretions or other innate immune pathways like TLR4, are complex (Man and Kanneganti, 2015). Recent literature focuses on AIM2 inflammasomes in *Ft* infections, are reviewed below.

Researchers have studied the role of the AIM2 inflammasome protein during *Ft* infection and immunization. AIM2 forms a complex with apoptosis-associated speck-like protein containing a CARD (ASC) to cleave pro-caspase-1 into caspase-1, which then activates pro-IL-1β and pro-IL-18 and induces gasdermin-D (GSDMD) to form pores in the cell membrane, allowing cytokine secretion. This process also triggers pyroptosis, a form of programmed cell death (Kumari et al., 2021) and is crucial for defending against intracellular pathogens, as shown in studies with *Ft* and *Brucella abortus* (Costa Franco et al., 2019). These molecular pathways are complex and interwoven with other innate immune signaling pathways, but their downstream effectors are distinct enough to require separate discussions.

Investigating *Ft*’s relationship with inflammasome mechanisms is challenging. Certain LVS mutants might be compromised structurally and lyse intracellularly, artificially increasing inflammasome signaling as host cytosolic DNA sensors detect the foreign DNA (Costa Franco et al., 2019). Singh et al. (2013) confirmed that immune responses to *Ft* differ between *in vivo* and *in vitro* systems, influenced by bacterial growth conditions and emphasized that not all *in vitro* findings may be relevant to tularemia pathogenesis in mammals. There is also evidence that ASC can function independently of caspase-1, bypassing canonical inflammasome activation (Cheong et al., 2020; Hughes et al., 2018). Additionally, murine BMDMs lacking ASC supported LVS growth similarly to those with functional ASC, indicating ASC’s limited impact on LVS growth. These findings were consistent across LVS strains with different mutations in the *Francisella* pathogenicity island (FPI). Interestingly, *MyD88*^−/−^ cells did not support significant replication of intracellular LVS, except for LVS strains with FPI mutations (Meyer et al., 2015). These redundant pathways downstream of ASC allow host immune cells to initiate the IFN-*γ*/IL-18 cascade. Further research is needed to understand how *Ft* evades or inhibits these redundancies in cell death pathways.

**Figure 3:**
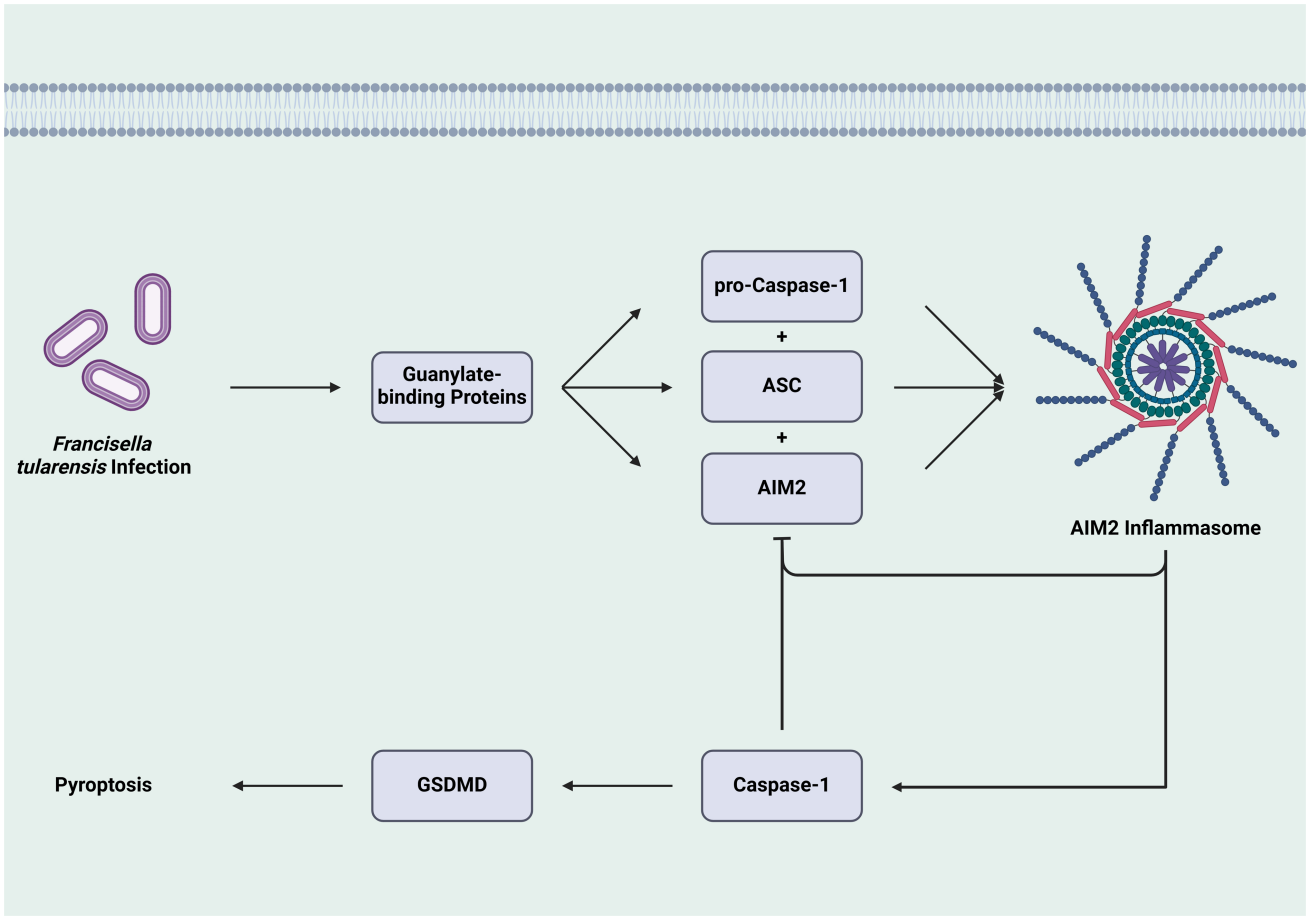
Feedback inhibition of inflammasome formation and caspase-1 activation. The construction of the AIM2 inflammasome through the combination of pro-caspase-1, ASC, and AIM, leads to the activation of caspase-1 and GSDMD, culminating in cell death *via* pyroptosis. However, the activity of the inflammasome and caspase-1 inhibit the formation of additional inflammasomes, which can attenuate the cell death pathway and allow *Ft*-infected cells to survive and contribute to the pathogen’s replication. While this pathway is specific to one mechanism, it is one that helps create an intracellular environment that facilitates *Ft* growth by preventing cell death (AIM2: absent in melanoma 2, ASC: adaptor molecule apoptosis-associated speck-like protein containing a CARD, GSDMD: Gasdermin D).

Within these pathways, multiple regulatory mechanisms exist, including negative feedback loops and protein complex formations. One such loop involves mature caspase-1 inhibiting AIM2 production. A 2013 study found that sublethal caspase-1 levels may prevent AIM2 from associating with the inflammasome adapter ASC, thus inhibiting AIM2 speck formation and subsequent pyroptosis (Juruj et al., 2013). This inhibition helps *Ft* evade the immune system and incubate within cells. Additionally, AIM2 activity blocks the stimulator of interferon genes (STING) pathway, which produces IFN-*β* and induces inflammasome activity. Therefore, as AIM2 inflammasome activity increases, it suppresses the upstream pathways that activate itself (Corrales et al., 2016; Figure 3).

The nonreceptor tyrosine kinase c-Abl impacts inflammasome signaling by interacting with IFN regulatory factor (IRF3), a key transcriptional regulator in IFN-*α* and -β production, which feeds into the pyroptosis pathway (Messaoud-Nacer et al., 2022). Typically, c-Abl is involved in cell cycle regulation, apoptosis, and antioxidant responses to hydrogen peroxide (H_2_O_2_) (Shaul, 2000; Cao et al., 2003). Though IRF3, c-Abl, and its relative Arg typically function independently, they are interconnected in inflammasome signaling. IRF3-mediated IFN-β production relies on binding and phosphorylation by c-Abl, and since AIM2 inflammasome induction depends on IFN-β, inhibiting c-Abl affects AIM2 activity. C57BL/6 mouse studies show that c-Abl is crucial for protection against LVS; inhibiting c-Abl with AMN-107 significantly reduced mRNA expression of IFN-β and AIM2, protein expression of IL-18, and survival rates. Small interfering RNA (siRNA) targeting c-Abl and Arg also decreased survival and expression levels, highlighting the complex regulatory interactions in the AIM2 inflammasome pathway and its role in *Ft* infections (Luo et al., 2019).

*Ft* can manipulate apoptosis pathways in human neutrophils. LVS robustly inhibits apoptosis, regulating 365 host genes related to apoptosis or cell survival. It downregulates the pro-apoptotic BCL2-associated X protein (BAX) and its mRNA 24 h post-infection, while sustaining the anti-apoptotic X-linked inhibitor of apoptosis protein (XIAP) for up to 48 h. Nuclear factor (NF)-κB markers are upregulated, promoting neutrophil survival (Schwartz et al., 2013). Despite these changes, proinflammatory signaling remains intact, consistent with an anti-apoptotic phenotype (Kobayashi et al., 2003). In 2015, it was further shown that LVS inhibits apoptosis markers like BAX, XIAP, and caspases −3 and − 9, and prevents apoptosis induced by R-roscovitine in neutrophils (McCracken et al., 2016).

IFNs can induce guanylate binding proteins (GBPs) in nearby host cells, which lyse the bacteria, exposing bacterial DNA to cytosolic sensors and triggering the AIM2/ASC inflammasome response. This results in the release of IL-1β and IL-18, intensifying the proinflammatory environment post-infection (Snyder et al., 2017). The cyclic GMP-AMP synthase (cGAS)-STING pathway can also be triggered by extracellular vesicles carrying bacterial DNA from nearby cells. These vesicles are produced via a STING-mediated pathway involving TANK-binding kinase 1 (TBK1) and multivesicular body subunit 12b (MVB12b) after *Ftt* infection. This cell-to-cell signaling activates the cGAS-STING pathway in target cells, leading to proinflammatory and inflammasome triggering. However, this process can aid intracellular pathogens by blocking IL-17A production, which is crucial for neutrophil expansion, thereby limiting aspects of the immune response (Nandakumar et al., 2019).

*Ft* evades the inflammasome by entering macrophages. Complement receptor 3 (CR3) binds to opsonized *Ft*, facilitating its uptake into macrophages (Hoang et al., 2018). Dai et al. (2016) found that opsonizing SCHU S4 before uptake reduces extracellular signal-regulated kinase (ERK) signaling, suppressing the inflammasome. This process upregulates Ras-specific GTPase-activating proteins (RasGAP), inhibiting the rapidly accelerated fibrosarcoma (Raf)-mitogen-activated kinase (MEK)-ERK pathway and suppressing innate immune pathways like TLR2. Opsonized *Ft* exploits these pathways to inhibit inflammasome activity, as shown by decreased mature caspase-1 and IL-18 secretion.

Inflammasome signaling molecules AIM2 and NLRP3 are not essential for vaccine-induced immunity against LVS. Alqahtani et al. (2021) found that *AIM2*^−/−^ and *NLRP3*^−/−^ C57BL/6 mice were fully protected against a lethal dose of LVS and showed normal antibody and cell-mediated responses. This suggests that AIM2 and NLRP3 are dispensable for vaccine-induced immunity against respiratory tularemia caused by *Ft*. However, it is unclear if this would hold true for a virulent strain of *Ft*. Overall, inflammasome activation is a complex and challenging aspect of tularemia infection, involving intricate regulatory mechanisms and interactions with different *Ft* strains. Identifying protective correlates is difficult, especially since early evidence suggests this pathway may be unnecessary for immune protection, as indicated by LVS.

#### 3.1.3 Toll-like receptors

Alongside the AIM2 inflammasome system, extracellular receptors like TLRs identify pathogens before they infiltrate cells. TLRs, working with key cytokines, trigger a rapid innate immune response by recognizing pathogen-associated molecular patterns. However, some pathogens evade this recognition, facilitating infection and replication. *Ftt* is particularly effective at evading the host immune system. A 2021 study showed that SCHU S4 does not trigger TLR2 or TLR4 responses in human embryonic kidney (HEK) 293 cells overexpressing these receptors (Amemiya et al., 2021). While LVS does not trigger TLR4, it robustly triggers TLR2. Unlike LPS from other Gram-negative bacteria, *Ft* lipopolysaccharide (LPS) does not interact with TLR4 to induce inflammation, contributing to its pathogenesis (Gunn and Ernst, 2007).

These innate immune interactions only partially explain how *Ft* interacts with its hosts. The pathogen-host interactions are highly complex, requiring significant crosstalk between the innate and adaptive immune systems. A recent study modeled these molecular interactions, identifying many potential targets for future therapies and preventative treatments (Miryala and Ramaiah, 2022). Despite progress in understanding these relationships, much work remains to translate these findings into medical countermeasures (MCMs) for humans.

#### 3.1.4 Suppression of ROS

Production of ROS is an important mechanism for control and elimination of intracellular pathogens; these ROS include microbicidal superoxide radicals (O_2_^−^), H_2_O_2_, and highly reactive hydroxyl radicals (HO^−^) produced from H_2_O_2_. To survive in this potentially hostile environment, *Ft* has evolved a variety of mechanisms to inhibit the activity of nicotinamide adenine dinucleotide phosphate (NADPH) oxidase, contributing to the organism’s pathology (Moreland et al., 2009; McCaffrey et al., 2010).

Several *Ft* proteins are differentially expressed under oxidative stress, notably those associated with translation processes, cellular stress, metabolism, and cell membrane proteins, in addition to changes in the *Igl* operon in the FPI of *Fth*. Affected proteins include ribosomal proteins, superoxide dismutase (Sod)B, acetyl CoA carboxylase, LpxD2, and IglA. These factors and others are differentially regulated in *Fth* so that it can both evade or suppress the host’s immune reaction while also providing necessary nutrients for its survival. Interestingly, the changes in proteins relating to oxidative stress were not induced if the host cells were previously primed with IFN-*γ* before infection, indicating that the early induction of IFN-γ’s targets more easily trigger an immune response from the host (Pavkova et al., 2013).

In a series of *in vitro* experiments, Newstead et al. (2014) infected murine MH-S alveolar macrophages and J774A.1 peritoneal macrophages with LVS or SCHU S4 to evaluate the relevance of nitric oxide (NO) induction in resistance to intracellular bacterial infection in a TLR-stimulated context. Cell monolayers were stimulated with bacterial (*E. coli*) LPS and IFN-γ. Importantly, these studies demonstrated that SCHU S4 appears to have a mechanism which prevents cellular nitrite production, and this mechanism is lacking in LVS. These studies demonstrate that LVS is not directly analogous to SCHU S4, and that caution should be exercised when extrapolating data gained from one strain to the other.

Ma et al. (2016) investigated the role of OxyR in the regulation of oxidative stress responses by LVS. These investigators found that OxyR of *Ft* regulates oxidative stress responses to promote resistance against ROS, thereby contributing to its intracellular survival and promoting its virulence in C57BL/6 mice. Proteomic analysis revealed that OxyR modulates the level of 128 proteins, indicating a broader regulatory role of OxyR in overcoming oxidative stress. Moreover, OxyR regulated the transcription of the primary antioxidant enzyme genes *ahpC* and *katG* by binding directly to their putative promoter regions. This finding was supported by later studies by Honn et al. (2017) using a double deletion mutant of LVS, *ΔoxyR/ΔkatG*, who demonstrated that *Ft* lacking OxyR and catalase were more susceptible to control by macrophages than WT *Ft*.

### 3.2 Antigen-presenting cells

In addition to identifying intracellular, molecular immune responses to Ft, our systematic review identified many cellular responses.

#### 3.2.1 Dendritic cells

**Figure 4:**
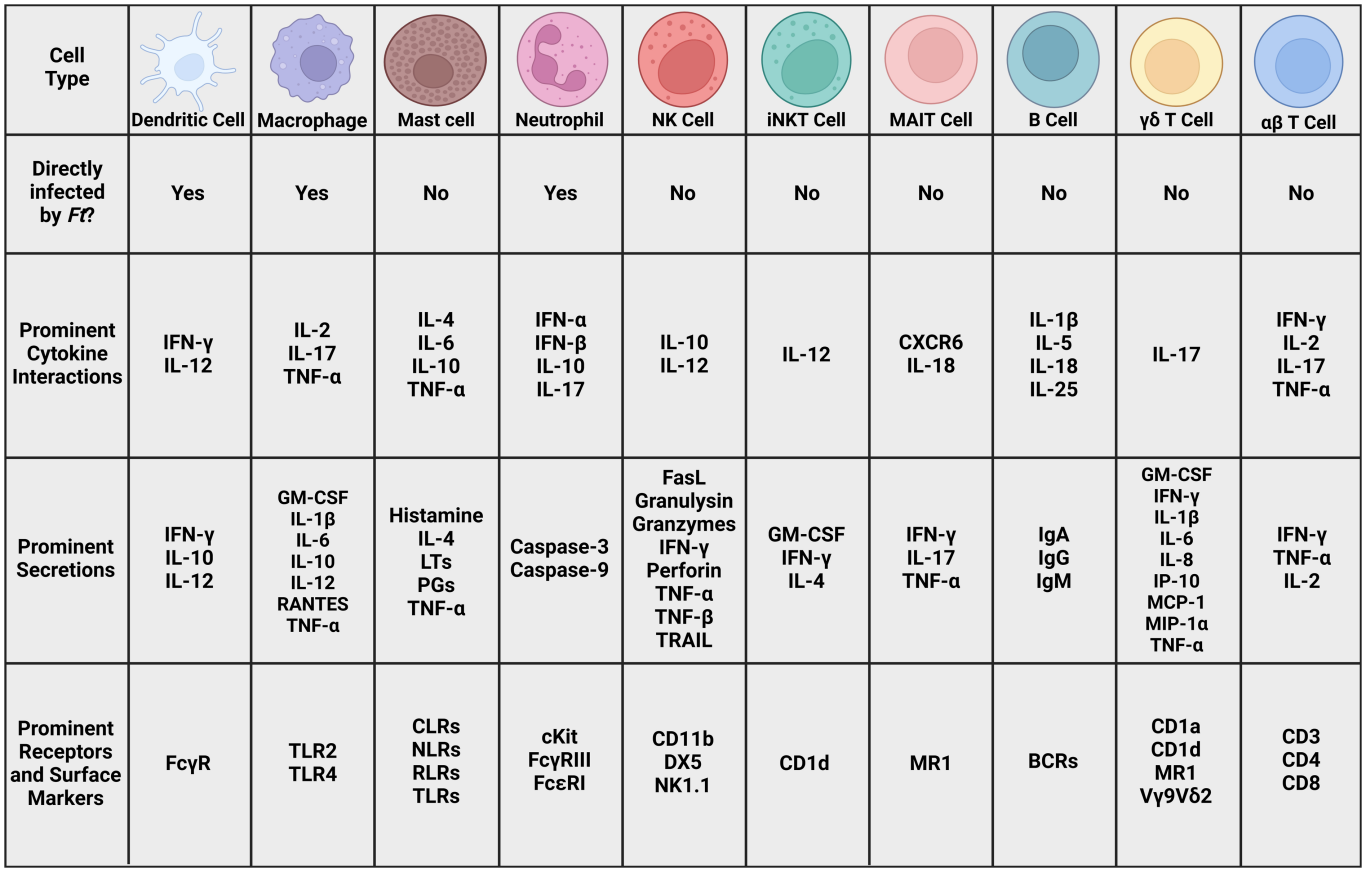
Prominent cell types in *Ft* literature. Many cell types of both the innate and adaptive immune system, as well as cells that bridge the gap between those systems. The cytokines, secretions, receptors, and surface markers involved in potentially recognizing and fighting tularemia infections. Given the complex interactions between these cells and signaling molecules, it is unlikely that any one will serve as a definitive correlate of protection. More research is necessary to elucidate these interactions and identify ones that could indicate vaccine efficacy (BCR: B cell receptor, CD: cluster of differentiation, CLR: C-type lectin receptor, CXCR: chemokine receptor, DX5: α_2_ integrin, FasL: Fas ligand, GM-CSF: granulocyte macrophage colony-stimulating factor, IFN: interferon, Ig: immunoglobulin, IL: interleukin, iNKT: innate natural killer T cell, IP: Interferon-gamma-inducible protein, LT: leukotriene, MAIT: mucosal-associated invariant T cell, MCP: monocyte chemoattractant protein, MIP: macrophage inflammatory protein, MR1: MHC class I-related protein, NK: natural killer cell, NK1.1: killer cell lectin-like receptor subfamily B member 1C protein, NLR: Nod-like receptor, PG: prostaglandin, RANTES: regulated on activation normal T cell expressed and secreted, RLR: RIG-I-like receptor, TRAIL: Tumor necrosis factor-related apoptosis-inducing ligand, TLR: toll-like receptor).

Various cell types in the innate immune system contribute to an appropriate response to a pathogen (Figure 4). One such cell type is the dendritic cell (DC), and *Ft* seems well-adapted to evade and suppress its immune mechanisms. Ireland et al. (2013) demonstrated that lipids isolated from SCHU S4 were sufficient to suppress an immune response in human DCs, notably suppressing the production of IL-12p40. This suppression extended to secondary exposure to LPS after the application of the SCHU S4 lipids. Additionally, these lipids had the same effect *in vivo* and prevented NF-κB from translocating to the nucleus of host cells in C57BL/6 J mice. In the same study, it was also shown that SCHU S4 was able to block the activity of IRF1 and IRF8, inhibiting the transcription of IL-12p40 (Ireland et al., 2013).

Administration of antigen (Ag)-pulsed DCs is an effective strategy for enhancing immunity to tumors and infectious organisms. Studies have also demonstrated that targeting antigens to Fc*γ* receptors (FcγR) on Ag-presenting cells can enhance humoral and cellular immunity *in vitro* and *in vivo*. Studies by Pham et al. (2014) using *Ft* demonstrated that targeting immunogens to Fc*γ*R *via* intranasal (i.n.) administration of inactivated *Ft* (i*Ft*) immune complexes enhanced protection against *Ft* challenge in C57BL/6 and Fc*γ*RIIB knockout (KO) mice on a C57BL/6 background. The authors sought to determine if *ex vivo* targeting of i*Ft* to FcγR on DCs would enhance the potency of i*Ft*-pulsed DCs administered i.n. Bone marrow-derived DCs (BMDCs) were pulsed *ex vivo* with i*Ft* or monoclonal antibody (mAb)-i*Ft* immune complexes. I.n. administration of these BMDCs enhanced humoral and cellular immune responses, as well as protection against LVS challenge. Increased protection correlated with increased i*Ft* loading on the BMDC surface due to Fc*γ*R targeting.

#### 3.2.2 Macrophages

*Ft* infection of macrophages has been well studied. The early interplay between *Ft* and the innate immune system is a key facet of infection, as macrophages are some of the earliest targets for infection by the bacterium. This is highlighted in a 2014 study in mice showing that *Fn,* SCHU S4, and LVS all preferentially infected alveolar macrophages (F4/80^high^, CD11c^high^, CD11b^mid^, DEC-205^mid^) in the first 4 h after inhalation in C57BL/6 mice (Roberts et al., 2014).

De Pascalis et al. (2018) investigated a macrophage co-culture assay to identify immune correlates for tularemia vaccines. They infected peripheral blood leukocyte (PBL)-derived macrophages taken from Fischer 344 rats with LVS and less protective LVS variants from LVS-immunized rats, then co-cultured these macrophages with PBLs from vaccinated rats. They found 22 genes were significantly upregulated in PBLs from LVS-vaccinated rats. Combining intramacrophage *Ft* growth control with specific gene expression levels [*IFN-γ*, *IL-21*, *nitric oxide synthase* (*NOS*)2, *lymphotoxin* *α* (*LTA*), *T-bet*, *IL-12rβ2*, and *chemokine ligand* (*CCL*)*5*] using multivariate analyses distinguished protected from non-protected rats with over 95% sensitivity and specificity. In 2022, they showed that more effective vaccines (e.g., *Ft* SCHU S4 Δ*clpB*) increased IFN-γ and nitric oxide levels while reducing bacterial levels in macrophages (De Pascalis et al., 2022). Further investigation into T cell-mediated infection control in macrophages and related cytokines is needed to develop candidates for immune correlates of protection.

In another study evaluating the role of macrophages in immunity provided by *Ft* vaccines (Babadjanova et al., 2015), C57BL/6 mice were vaccinated with paraformaldehyde-inactivated *Ft* in a prime-boost strategy. Peritoneal macrophages from immunized mice produced more IL-12p70 and TNF, and less IL-10 than unvaccinated mice. The addition of a mAb to the inactivated *Ft* to enhance macrophage targeting further enhanced IL-12p70 and TNF production and further reduced IL-10 production. IL-10 production from macrophages was shown to attenuate pro-inflammatory cytokine production.

A 2016 study utilized an *in vitro* model to simulate macrophages in the human liver by co-culturing HepaRG hepatocytes and BMDMs. The application of LVS, WT *Fth*, and *Ftm* caused macrophage cell death and production of TNF, IL-6, IL-1β, and fractalkine (Rennert et al., 2016). This model may have utility in examining Kupfer cell (i.e., liver resident macrophages) contributions to *Ft*-specific immunity.

Rabadi et al. (2016) found that macrophages infected with LVS produced little IL-6 and TNF and possessed potent anti-inflammatory capabilities. Live, but not UV-killed, LVS suppressed IL-6 and TNF production, consistent with previous SCHU S4 studies (Gillette et al., 2014). *Ft* SodB and mitogen-activated protein kinase (MAPK) contributed to this suppression by preventing the activation of redox-sensitive MAPK signaling, NF-κB signaling, and pro-inflammatory cytokine production through inhibition of ROS accumulation in infected macrophages.

Golovliov et al. (2016) studied NO as a predictor of *Ft* vaccine efficacy using an *in vitro* co-culture model with murine BMDMs and splenocytes from BALB/c and C57BL/6 mice immunized with LVS or the Δ*clpB* attenuated strain of *Ft*. Similar to De Pascalis et al. (2018) they found that nitrite levels in the supernatants of splenocyte-BMDM co-cultures infected with LVS strongly correlated with growth inhibition and levels of IFN-*γ*, granulocyte-macrophage colony-stimulating factor (GM-CSF), IL-6, IL-10, IL-12p40, and CCL5. Δ*clpB*-immune splenocytes produced more nitrite than LVS-immune splenocytes. When SCHU S4 was the infecting agent, nitrite levels also correlated with *Ft* growth inhibition, with Δ*clpB*-immune splenocytes showing higher nitrite levels than LVS-immune splenocytes. These findings suggest a link between cytokine and nitrite production and growth suppression by both LVS and SCHU S4. Similar results were observed in studies using Fischer 344/IcoCrl rat macrophages and splenocytes (Lindgren et al., 2020). Demonstrating this correlation in human cell cultures would be important, though technically challenging to adapt for vaccine development.

Macrophages stimulated with IFN-γ can resist *Ft* infection *in vitro*. To determine if this protection requires macrophages receptive to stimulation, Steiner et al. (2017) used alveolar macrophage depletion in WT C57BL/6, BALB/c, and IFN-γ insensitive mice (MIIG model) with sublethal pulmonary LVS infection. Both macrophage depletion and IFN-γ insensitivity worsened the infection. While exogenous IL-12 usually provides protection in mice, this was not seen in MIIG mice, indicating IL-12’s effects are not directly through macrophages. MIIG mice also showed reduced neutrophil recruitment to the lungs after LVS infection.

Richard et al. (2016) discovered that IFN-*β* enhances macrophage resistance to LVS infection by increasing TLR2’s response to *Ft*, leading to the production of pro-inflammatory cytokines like IL-6. This enhanced innate recognition and TLR-dependent transcriptional response were observed in primary human monocyte-derived macrophages and primary murine peritoneal macrophages, but not in murine BMDMs. The type I IFN-enhanced response works synergistically with TLR2 transcriptional responses and is partially TLR2-independent but strictly MyD88-dependent. These findings differ from other macrophage studies where IFN-β reduces pro-inflammatory cytokine production, complicating the use of cytokines to measure immune responses to *Ft*.

All these studies suggest that macrophage cytokine production, as influenced by other immune cell types and *Ft* virulence factors, contribute to *Ft* infection outcomes. Macrophage cytokine production, particularly in co-culture with immune lymphocytes has great potential for the development of an immune correlate of protection, but additional work is required.

#### 3.2.3 Dendritic cell cytokines

IFN-γ production in response to *Ft* infection is known to occur by NK and T cells, but the production of this cytokine by DCs is less-studied. De Pascalis et al. (2020) demonstrated substantial production of IFN-*γ* by DC isolated from wild-type (WT) C57BL/6 and *Rag1* KO mice infected with LVS, as well as hybrid NK-DC, from LVS-infected *Rag1* KO mice. This indicates that DC produced IFN-*γ* independently of B or T cells. These authors demonstrated that the numbers of conventional DC producing IFN-γ increased progressively over the course of 8 days of LVS infection; in contrast, the numbers of conventional NK cells producing IFN-γ, which represented about 40% of non-B/T IFN-γ-producing cells, peaked at Day 4 after LVS infection and declined thereafter. This pattern was similar to that of hybrid NK-DCs. To further confirm IFN-γ production by infected cells, DC and neutrophils were sorted from naive and LVS-infected mice and analyzed for gene expression. Quantification of LVS by polymerase chain reaction (PCR) revealed the presence of *Ft* DNA not only in macrophages but also in highly purified, IFN-*γ* producing DCs and neutrophils. Finally, production of IFN-*γ* by infected DCs was confirmed by immunohistochemistry and confocal microscopy. Notably, IFN-γ production patterns similar to those in wild type C57BL/6 J mice were observed in cells derived from LVS-infected *TLR2*, *TLR4*, and *TLR2*x*TLR9* KO mice, but not from *MyD88* KO mice. These data add to the complexity of IFN-γ as an indicator of a specific immune response, as IFN-γ can come from multiple sources, dependent and independent of a memory immune response.

IL-12 is produced by DCs, macrophages, and B cells following infection with pathogenic bacteria and stimulates IFN-*γ* production by T cells. IFN-γ also stimulates IL-12 in antigen-presenting cells, creating a feedback loop that facilitates Th1 cell differentiation; the Th1 response is important for responding to intracellular pathogens including *Ft*. IL-12 can also facilitate the production of IFN-γ by NK cells (Vignali and Kuchroo, 2012). IL-12 is often designated IL-12p70, since it is a heterodimeric cytokine comprising p40 and p35 subunits.

It has been known for more than two decades that infection with *Ft* in mice results in a rapid induction of IL-12, along with TNF-*α* and IFN-*γ* (Stenmark et al., 1999). To better understand the role of IL-12 in tularemia, Elkins et al. (2002) employed BALB/cByJ and C57BL/6 J murine models of IL-12 deficiency (*p40* KO mice, *p35* KO mice, and mice treated with IL-12-neutralizing antibodies) infected with LVS. *p40* KO mice and mice treated *in vivo* with anti-IL-12 antibodies survived large doses of primary and secondary LVS infection but never cleared bacteria, instead resulting in a chronic infection. *p35* KO mice survived large doses of primary sublethal LVS infection as well as maximal secondary otherwise lethal challenge. LVS-immune lymphocytes from WT mice produced large amounts of IFN-γ, while *p35* and *p40* KO lymphocytes produced much less. The authors surmised that while IL-12p70 stimulation of IFN-γ production may be important for bacteriostasis, it is not necessary for controlling intracellular bacterial growth and for clearance of primary or secondary LVS infection. Similar results were more recently found in C57BL/6 J mice by Mittereder et al. (2023).

The role of IL-12 in inducing protective immunity in the lungs of BALB/c and C57BL/6 mice against i.n. infection with LVS was further investigated by Duckett et al. (2005). They found that IFN-γ and IL-12 were strictly required for protection, since mice deficient in IFN-γ, IL-12p35, or IL-12p40 all succumbed to LVS doses that were sublethal for WT mice. They further showed that exogenous IL-12 treatment 24 h prior to infection with a lethal dose of LVS significantly decreased bacterial loads in the lungs, livers, and spleens of both WT BALB/c and C57BL/6 mice and allowed the animals to survive infection; this protection was not observed in IFN-γ-deficient mice. The resistance to LVS induced by IL-12 was also observed in NK cell-deficient mice, although not in *CD8*^−/−^ mice, suggesting that this mechanism is at least partially dependent upon the expression of IFN-*γ* and CD8^+^ T cells.

Using *in vitro* cultures of *Ft*-infected, human monocyte-derived DCs, Bauler et al. (2011) found that whereas LVS infection quickly induced the production of several cytokines including IL-12p40, SCHU S4 infection did not result in production of inflammatory cytokines. This difference between LVS and SCHU S4 was noted even though both strains proliferated in culture to equivalent degrees. Further evaluation revealed SCHU S4 induced IFN-*β*, which in turn blocked IL-12p40 production in DCs. Subsequent studies by Ireland et al. (2013) revealed that this activity was mediated by lipids in SCHU S4 (and not LVS) that inhibited IRF1 and IRF8. *Nota bene*: This clear differential effect between virulent and non-virulent strains of *Ft* suggests that caution should be exercised when extrapolating results from historical LVS research to more virulent strains.

### 3.3 B cells, antibodies, and associated cytokines

Antibodies are a key component in control of and protection from many microbial pathogens; however, our understanding of the precise role of antibodies during the immunological response to *Ft* has a long and often contradictory history. The antibody response to *Ft* exposure is complex, involving immunoglobulin (Ig)A, IgM, and IgG, and includes the participation of both antigen-specific and natural antibodies (Kubelkova and Macela, 2021; Kubelkova et al., 2021). It should also be noted that several studies have shown that transfer of immune sera or monoclonal antibodies to naive animals did not provide protection against LVS (Rhinehart-Jones et al., 1994; Narayanan et al., 1993; Dreisbach et al., 2000). Much of the response is specific to *Ft* LPS (Kirimanjeswara et al., 2008), especially the O-antigen (O-polysaccharide component of the LPS complex).

Injection of LVS membrane protein fractions after infection has yielded elevated IgM and IgG in BALB/c murine sera, which, combined with gentamicin regimens, has been efficacious against *Ft* SCHU S4 infection (Chandler et al., 2015). Secondary computational analysis of the membrane fractions identified a subset of LVS surface proteins likely to elicit immunogenic responses, however it is unclear exactly how these factors can be used for prophylactic or post-exposure immunization (Chandler et al., 2015) or if specific antibody epitopes could be used to predict survival outcomes.

Richard et al. (2014) generated antisera by immunizing C57BL/6 mice with LVS vesicles. Those antisera appeared to confer limited and passive protection against challenge with LVS. This novel vaccine approach used synthetic nanoparticles made from catanionic surfactant vesicles functionalized by the incorporation of either LVS or SCHU S4 components. Vesicles which did not express *Ft* components partially protected against LVS yet failed to protect against SCHU S4. In contrast, immunization with LVS vesicles (LVS-V) fully protected C57BL/6 mice against intraperitoneal (i.p.) LVS challenge, while immunization of mice with either LVS vesicles or SCHU S4 vesicles partially protected them against an i.n. SCHU S4 challenge and significantly increased the mean time to death for non-survivors. LVS-V immunization, but not immunization with empty vehicles, elicited high levels of IgG against non-LPS epitopes that were increased after LVS challenge and significantly increased early cytokine production.

Many studies of the role of antibodies in control of *Ft* have focused on IgM and IgG (Kubelkova and Macela, 2021). IgA, the most abundant antibody isotype in mucosal tissues, may have importance in *Ft* lung infections. *IgA*^−/−^ mice vaccinated with UV-inactivated LVS plus exogenous IL-12 were not protected from subsequent live LVS challenge, but vaccinated C57BL/6 mice with *IgA* were protected (Baron et al., 2007). This suggests that in the LVS-sensitive mouse model, IgA may play a role in protection. Kubelkova and Macela (2021) provide a more comprehensive review of the role of antibodies in protection from *Ft*. However, we found additional points of interest regarding B1b cells.

#### 3.3.1 B1b cells

Several investigators have explored the protective function of B1a cells. Studies in which *Ft*-LPS was administered to various mouse models (Cole et al., 2009) found that all animals pre-treated with 100 ng *Ft*-LPS were protected from LVS infection, whereas control mice all died. This protection was long-lived, up to 72 days post-treatment. B cells were required, as demonstrated by the lack of protection against LVS challenge following LPS vaccination in μMT mice (deficient in B cells) and JhD mice (IgH chain deletion). Studies with X-linked immunodeficiency (XID) mice (severely deficient in B1a cells) resulted in animals losing *Ft*-LPS-induced protection, demonstrating that B1a cells are required for this model. This was further confirmed by noting that *Ft*-LPS pretreatment induced large numbers of antigen-specific B1a cells, which differentiated into anti-*Ft*-LPS antibody-secreting plasma cells. Subsequent studies by this group (Cole et al., 2011) with C57BL/6 *TLR2*^−/−^ mice suggested that *Ft*-LPS-mediated protection against LVS infection requires two discrete events, the first being the production of *Ft*-LPS-specific antibody and the second being TLR-mediated macrophage activation.

Recent evidence suggests that innate lymphoid cells (ILCs) of the second (lung mucosal) subset (ILC2s) can contribute to the activation of B1 lymphocytes after C57BL/6 murine immunization with LPS isolated from LVS. This process, traditionally thought to be carried out predominantly by TLRs and MyD88, can operate independently through the secretion of interleukin (IL)-25 by ILC2s. IL-25, a member of the IL-17 family, then stimulates other ILC2s to release IL-5, inducing the migration and differentiation of B1 cells to antibody-secreting cells. This response was seen only with LPS isolated from LVS, not with LVS infection itself (Barbosa et al., 2021).

#### 3.3.2 IL-1β/IL-18

Del Barrio et al. (2015) found that during LVS infection, antibodies were produced by B1a cells that decreased susceptibility in a cytokine-dependent fashion. C57BL/6, *IL-1rβ*^−/−^, or *IL-18*^−/−^ mice infected i.n. with LVS exhibited greater susceptibility to LVS than controls, with mean time to death and bacterial burdens in *IL-18* knockout (KO) mice greater than those seen in *IL-1β* KO mice. The increased susceptibility was not observed with *IL-1rα*^−/−^ mice. Mice deficient in IL-18 quickly succumbed to infection with LVS, but this was found to be mitigated by administration of exogenous IFN-*γ*. On the other hand, mice deficient in IL-1β were able to control the infection initially but eventually died from the infection, and this could not be mitigated by exogenous IFN-*γ*, indicating that IL-1β and IL-18 operate differentially in response to *Ft*. The IL-1β-deficient mice were found to have significantly reduced serum levels of IgM antibodies specific for *Ft* LPS. In control mice, these antibodies promoted bacterial agglutination and phagocytosis and were protective in passive immunization experiments. These antibodies were determined to be produced by B1a cells, which were significantly decreased in the spleen and peritoneal cavity of infected IL-1β-deficient mice. The authors concluded that IL-1β and IL-18 activate non-redundant protective responses against tularemia and identify an essential role for IL-1β in the rapid generation of pathogen-specific IgM by B1a cells. While B1b cells may play an important role in murine LVS infections, it is important to note that mouse lung infections may not be reflective of other species (most importantly humans).

### 3.4 T cells and associated cytokines

T cells have long been known to contribute to protection against tularemia (Tarnvik, 1989) and the production of cytokines from T cells, such as IFN-*γ*, has also long been known as a key component of this protection (Messaoud-Nacer et al., 2022; Shaul, 2000). In studies by Sjostedt et al. (1994), the responses to four *Francisella* antigens were shown to be confined mostly to the CD45RO^+^ memory T-cell subset. To characterize further the phenotype of the responding cells, purified CD4^+^ and CD8^+^ T cells were stimulated with the antigens. CD4^+^ T cells, but not CD8^+^ T cells, proliferated and produced IFN-*γ*. However, when conditions permitted or simulated CD4^+^ T cell help, CD8^+^ T cells responded similarly to CD4^+^ T cells. There was a direct quantitative correlation between the proliferative response of CD4^+^ and CD8^+^ T cells and their production of IFN-γ. In conclusion, both CD4^+^ and CD8^+^ T cells of LVS-vaccinated humans respond with proliferation to various protein antigens of *Ft*, and the proliferative response was strictly associated with IFN-γ production.

Roberts et al. (2016) evaluated the importance of engaging CD4^+^ cells by generating *Ft* strains expressing well-characterized lymphocytic choriomeningitis virus (LCMV) epitopes and using these epitopes to track the T cell response. They found that T cells from vaccinated C57BL/6 J mice could be isolated and could control *Ft* replication in macrophages cultured *in vitro* and that prolonged survival time was related to the major histocompatibility complex (MHC) haplotype capable of presenting the LCMV epitope to T cells. Furthermore, the T cells produced IFN-*γ*, TNF, and IL-2, which adds to the literature showing that T cells producing multiple cytokines are protective against tularemia.

De Pascalis et al. (2012) found that *T-bet*, a T cell transcription factor, was strongly upregulated in immune lymphocytes. T-bet (also known as TBOX21 or TBX21) is a transcription factor specific to IFN-*γ*-producing T cells (Szabo et al., 2002; Lazarevic and Glimcher, 2011). T-bet has been increasingly recognized as having multiple functions, particularly as a bridge between the innate and adaptive immune responses (Lazarevic et al., 2013) and as a regulator of mucosal immunity (Mohamed and Lord, 2016). Melillo et al. (2014) found that intradermal (i.d.) or i.n. infection of C57BL/6 J with LVS was lethal at a two log-lower dose in T-bet deficient mice by both i.d. and i.n. dosing. T-bet deficient mice also exhibited significantly increased bacterial burdens in the lung and spleen. These differences were associated with changes in cytokine levels (IFN-*γ*, TNF, and IL-17) and recruitment of B cells, T cells, neutrophils, macrophages, DCs, and NK cells to the lungs. LVS-vaccinated *T-bet*-KO mice survived lethal LVS i.p. challenge but not i.n. challenge, regardless of the route of vaccination (i.d. or i.n.). Immune T lymphocytes from the spleens of i.d. LVS-vaccinated WT or *T-bet*-KO mice controlled intracellular bacterial replication in an *in vitro* co-culture system (described further in the macrophage section above), but cultures with *T-bet*-KO splenocyte supernatants contained less IFN-*γ* and increased amounts of TNF-*α*. *T-bet*-KO lung lymphocytes were greatly impaired in controlling intramacrophage growth of LVS. The authors concluded that “...T-bet represents a true, useful correlate for immunity to LVS”; whereas this may be accurate regarding protection against LVS, it remains uncertain whether this is true for virulent strains of *Ft*.

Numerous studies have investigated the cytokine response following naturally acquired infection with *Ft*, as well as the immune response induced by various tularemia vaccines (Lindgren et al., 2020; Eneslatt et al., 2012; Cunningham et al., 2020; Goll et al., 2020; Lindgren et al., 2023). Infection with *Ft* has been shown to induce hypercytokinemia (“cytokine storm”) in which infection results in an uncontrolled upregulation of various inflammatory cytokines with subsequent tissue damage and pathology (Mares et al., 2008; D’Elia et al., 2013; Ramos Muniz et al., 2018). Thus, understanding cytokine responses in *Ft* infection and immunization is crucial. Some of the cytokines that have been studied in the context of immunity to tularemia are discussed below.

#### 3.4.1 Type II IFNs

Arguably, IFN-*γ* has been assessed more than any other cytokine in the context of tularemia and responses to vaccination. IFN-γ is a pivotal cytokine with multiple functions and is a key mediator of both innate and adaptive immunity (Kak et al., 2018; Mezouar and Mege, 2020). Production of IFN-*γ* following exposure to *Ft* antigens, either by natural infection or immunization, is a characteristic cellular response to *Ft*. In studies by Karttunen *et al*. (Karttunen et al., 1987), IL-2 and IFN-γ production were evaluated in subjects naturally infected 2 years previously, subjects vaccinated with LVS 5–6 years previously, and control subjects with no history of infection or vaccination. Subjects with a history of naturally acquired infection synthesized more DNA in both whole-blood and mononuclear cell cultures and secreted more IL-2 and IFN-γ than control subjects did. Vaccinees exhibited somewhat lower responses than the naturally infected subjects did, but in terms of DNA synthesis and IL-2 secretion responded in a manner similar to that of this group, with respect to receptor positivity and IFN-γ secretion.

Given the importance and variability of IFN-γ’s actions in the immune response to *Ft*, Parsa et al. (2008) hypothesized that *Ft* could interfere with IFN-γ downstream intracellular signaling. These authors demonstrated that *Ft* suppresses IFN-γ-induced signal transducer and activator of transcription (STAT) 1 expression and phosphorylation in both human and murine mononuclear phagocytes, and that this suppressive effect was independent of phagosomal escape or replication and was mediated by a heat-stable and constitutively expressed bacterial factor. An analysis of the molecular mechanism of STAT1 inhibition indicated that expression of suppressor of cytokine signaling (SOCS)3, an established negative regulator of IFN-γ signaling, was highly upregulated during infection and suppressed STAT1 phosphorylation. Functional analyses revealed that this interference with IFN-γ signaling was accompanied by the suppression of IFN-γ-induced protein (IP)-10 production and inducible NOS induction, resulting in increased intracellular bacterial survival. It remains unclear if vaccination impacts this *Ft*-mediated IFN-*γ* signaling disruption and if it contributes to immune correlates.

#### 3.4.2 IL-17

IL-17 is a key cytokine for host protection against mucosal infections. The IL-17 family comprises six members (IL-17A to IL-17F) that mediate their functions through several receptors. The most studied IL-17 family member is IL-17A, generally referred to simply as IL-17. IL-17 is produced primarily by IL-17-secreting CD4^+^ T (TH17) cells; it is also produced by CD8^+^ T cells, gammadelta (γδ) T cells, NK cells, invariant NK T cells, mucosal-associated invariant T (MAIT) cells, mast cells, and others. Whereas T cell receptor (TCR) activation is key for IL-17 production by CD4^+^ and CD8^+^ T cells, IL-17 production by innate immune cells is primarily driven by inflammatory cytokines, especially IL-1β and IL-23 (Mills, 2023). One of the functions of IL-17 during *Ft* infection is regulation of the Th1 immune response. Studies by Lin et al. (2009) in C57BL/6 mice infected intratracheally with LVS demonstrated that IL-17 induced IL-12 production in DCs and thus mediated Th1 responses. In addition, they demonstrated that IL-17 also induces IL-12 and IFN-γ production in macrophages and mediated bacterial killing.

Murine studies utilizing C57BL/6 and BALB/c strains with sub-lethal inhalational doses of LVS revealed IL-17 in lavage fluids of infected lungs with pulmonary γδ and Th17 cells as the IL-17 sources. IL-17 production appeared early during infection and preceded the peak of immune activation, afterward subsiding. The investigators demonstrated that exogenous airway administration of IL-17 (or IL-23, which leads to the development and activity of Th17 cells) had a limited yet consistent effect of delaying the onset of death. The protective role for IL-17 against LVS was directly demonstrated by *in vivo* neutralization of the cytokine with anti-IL-17 antibodies, resulting in a fatal disease (Markel et al., 2010). Subsequent studies with SCHU S4, however, clearly demonstrated that IL-17 is not involved in resolution of infection (Skyberg et al., 2013). Skyberg et al. (2013) (again using C57BL/6 and BALB/c strains) were able to replicate the protective role of IL-17, with *IL-17*^−/−^ mice less able to control LVS replication in the lung and spleen, but SCHU S4 infected *IL-17*^−/−^ mice having similar numbers of bacteria as WT. Importantly, *IL-17*^−/−^ mice orally vaccinated with LVS were equally protected from a subsequent SCHU S4 challenge, relative to WT mice. These data caution against the overinterpretation of studies based on LVS alone.

Studies (Roberts et al., 2014) have shown that a secondary infection in mice immunized with LVS or LVS *clpB* did not induce an IL-17 response that was different from control mice, suggesting that the IL-17 cytokine is not required for protection during secondary infection (i.e., immunological memory). These investigators followed up by treating immunized (LVS and LVS *clpB*) mice with anti-IL-17 antibodies, and then challenging the animals with LVS i.n. The mice were subsequently evaluated for microbial burden and weight loss and were found to not be significantly different from controls. Therefore, while it has been shown that IFN-γ continues to play a major role during a secondary (memory) response, IL-17 was not important. Additional studies have shown the ability of the immunoregulatory cytokine IL-10 to downregulate the activity of IL-17 during *Ft* infection of C57BL/6 and BALB/c mice, apparently preventing an over-accumulation of neutrophils and thus limiting the inflammatory response (Slight et al., 2013; Metzger et al., 2013). These studies, together with the findings of Skyberg et al. (2013) indicates that while IL-17 figures prominently in tularemia immune responses (though less so for SCHU S4), its role is limited in memory immune responses.

#### 3.4.3 IL-6

IL-6 is a pleiotropic cytokine with key activities in regulating immunity and inflammation (Tanaka et al., 2014). Its production is known to vary during *Ft* infection as well as during immunization with LVS. For example, Krakauer and colleagues (Krakauer, 1995) found that in human volunteers immunized with LVS, circulating levels of IL-6 (along with TNF) were higher in humans with a positive antibody response to the vaccines compared with non-responders.

In more recent studies, Chiavolini et al. (2008) infected BALB/c mice with LVS and evaluated post-infection cytokine levels in serum, homogenized lung, and homogenized spleen tissues at various time points. Cytokines were measured from both moribund and surviving animals, and levels were compared. Macrophage inflammatory protein (MIP)-2, monocyte chemoattractant protein (MCP)-1, and IL-6 levels were elevated above baseline in lungs and spleens of moribund mice, while in surviving mice, levels were similar to uninfected controls. The splenic increases in MCP-1 and IL-6 levels were highly significant; an IL-6 increase in serum was noted, although this was not statistically significant. The authors cited the previous observation that IL-6 is a known marker of sepsis. Interestingly, in the same year, Ashtekar et al. (2008) demonstrated that purified *Ft* heat shock protein DnaK induced production of IL-6 and other proinflammatory cytokines in mouse-derived DCs. In studies using *in vitro* recall stimulation of PBLs from naive, immunized, or convalescent human donors with purified antigen from either LVS or SCHU S4, Eneslatt et al. (2012) demonstrated that a panel of cytokines including IL-6 was able to discriminate between naive and immune donors; moreover, IL-6 was able to discriminate between the immunized and convalescent donors. Using an *IL-6* KO C57BL/6 mouse strain, Kurtz et al. (2013) demonstrated that IL-6 is essential to primary resistance to infection (either i.d. or i.n.) with LVS; this protection did not appear to be due to altered splenic immune cell populations during infection or decreased serum antibody production, as *IL-6* KO mice had similar compositions of each compared to WT mice. However, studies by Laws et al. (2013) using the virulent SCHU S4 suggested that IL-6 does not have any effect on the progression of this disease. Clearly, continued effort examining IL-6 in the context of *Ft* infections is warranted.

### 3.5 Additional cell types

Thus far, we have focused on the major lymphocyte and antigen-presenting cell types. However, many other cell types have been investigated in response to *Ft* infection and/or vaccination. We discuss recent findings in these cells next.

#### 3.5.1 Innate T cells

We have described the recent work in conventional alpha beta (αβ) T cell subsets. While conventional αβ T cells recognize a broad range of peptide antigens typically presented by MHC I and II complexes, enabled through their highly diverse TCR arrangement. Conversely, innate T cells are a heterogeneous group of αβ and *γ*δ T cells that respond rapidly (<2 h) upon activation and which recognize foreign/self-lipid presented by non-classical MHC molecules such as CD1a, CD1d, and MHC class I-related protein (MR1). Innate T cells are activated during the early stages of bacterial infection and act as a bridge between the innate and adaptive immune systems (Gao and Williams, 2015; Harly et al., 2022). Three types of innate T cells, γδ T cells, MAIT cells, and Invariant Natural Killer T (iNKT) cells have been shown to have roles in tularemia. MAIT and iNKT cells are both restricted αβ T cells. Except for iNKT cells, the role of these various populations in tularemia and vaccines against *Ft* has been evaluated to varying degrees, as is discussed below.

#### 3.5.2 γδ T cells

Although γδ T cells constitute only a few percent of the circulating lymphocyte population, they represent up to 20% of lymphocytes resident in the lungs (Cheng and Hu, 2017). In one of the earliest examinations of the role of γδ T cells in tularemia, Poquet et al. (1998) measured the ratio of blood circulating αβ and γδ T cells in tularemia-infected and uninfected humans. There was a dramatic increase in γδ T cells in infected patients. This increase was due primarily to the Vγ9Vδ2 phenotype, a γδ T cell subset with specificity for phosphoantigens, and found in primates but not rodents or most other mammals. This was mirrored by *in vitro* studies in which PBLs were co-cultured with *Ft* extract, with similar expansion of the Vγ9Vδ2 cells with extracts from either LVS or virulent strains of *Ft*. Interestingly, a similar expansion was not observed when PBL from individuals immunized with LVS were tested *in vitro*. These findings were expanded on by Kroca et al. (2000), who found that the expansion of Vγ9Vδ2 did not occur within the first week of infection; however, after the first week of infection the percentage of Vγ9Vδ2 increased dramatically and remained higher than respective controls for 18 months before returning to control levels by 24 months. Rowland et al. (2012) expanded on these findings by culturing human Vγ9Vδ2 T cells with SCHU S4-infected macrophage cells. They found that Vγ9Vδ2 T cells controlled SCHU S4 growth and increased culture levels of GM-CSF, IFN-γ, IL-1ꞵ, IL-6, IL-8, IP-10, MCP-1, MIP-1ɑ, and TNF.

Challenges limit the utility of γδ T cells, namely that the various subsets of γδ T cells that exist in mice and humans do not necessarily have overlapping identifying markers or functionality (Qu et al., 2022). The role of murine *γ*δ T cells was further explored by Henry et al. (2010). In these studies, BALB/cJ mice deficient for type I IFN receptor (*IFNAR1*^−/−^) were infected i.n. with SCHU S4. *IFNAR1*^−/−^ mice presented higher levels of IL-17A in the spleen, but levels were similar to the WT in other organs. This suggests that type I IFNs suppress IL-17A, potentially through changes in *γ*δ T cell activity. However, additional studies are recommended to confirm this connection. Overall, there is promise in γδ T cell activity, however, their limited T cell repertoire makes it unclear how they contribute to *Ft*-specific immunity.

#### 3.5.3 Invariant natural killer T cells

Mucosal surfaces, including the lungs, are populated by NKT cells, thus representing an important cellular milieu for interacting with *Ft*. Given their immunoregulatory activity, NKT cells have important roles to play in both immunity and pathology within the lungs (Paget and Trottein, 2013; Kumar and Delovitch, 2014). Perhaps because NK cells and NKT cells are so similar in their function, the few studies that reference NKT cells in the context of tularemia also include NK cells. Also, a major impediment to evaluating the potential role of iNKT cells is posed by the difficulty of experimentally differentiating the roles of NK and iNKT cells, given the high degree of similarity between them. Antibody-mediated depletion is impractical since there are currently no known iNKT-specific surface receptors; this leaves the use of genetically modified mice, in particular *CD1d*^−/−^ mice. In the only study we identified with focus on NKT cells, Hill and colleagues (Hill et al., 2015) infected WT C57BL/6 and *CD1d*^−/−^ mice with LVS and evaluated progression of disease. By 4 days post infection (dpi) both WT and *CD1d*^−/−^ mice began exhibiting signs of disease, including weight loss and ruffled fur. By dpi 5–6, WT mice continued to lose more weight and showed more severe outward signs of disease as indicated by clinical score. While the WT mice continued to lose weight, by dpi 7 nearly all *CD1d*^−/−^ mice began to recover and a significantly lower percentage of *CD1d*^−/−^ mice succumbed to LVS infection relative to the WT mice. By dpi 14, fewer than 50% WT and almost all *CD1d*^−/−^ mice regained weight, showed no signs of disease, and survived the infection. The authors concluded that NKT cells exacerbate pneumonic tularemia. To confirm this finding, the investigators infected V*α*14^tg^ mice, which have increased iNKT cell activity. These animals were indeed found to have increased susceptibility to infection with LVS. The authors further found that LVS infection preferentially recruits NKT cells to the lung interstitium. Investigations into the role of NKT cells in infection after vaccination may be of value for further elucidating the complex immune response to *Ft*.

#### 3.5.4 MAIT cells

MAIT cells are a subset of innate-like T cells that express an evolutionarily conserved T cell receptor α chain that is restricted by the non-polymorphic MR1. They are activated by microbial riboflavin metabolite-derived antigens, and this distinguishes them from all other *αβ* T cells (Meierovics and Cowley, 2016; Legoux et al., 2020). In humans, the highest concentration of MAIT cells is found in the blood and liver, with approximately 70% of these cells exhibiting a CD8^+^ phenotype and 15% with a double-negative phenotype. However, MAIT cells are also enriched in the mucosal tissues, with between 40 and 80% having the double-negative phenotype (Nel et al., 2021).

MAIT cells have been extensively studied in tularemia. In a C57BL/6 murine model of pulmonary LVS infection, Meierovics et al. (2013) found that MAIT cells expanded significantly in the lungs during the acute infection phase and accumulated there after peak expansion in the late phase. These cells produced IFN-*γ*, TNF-α, and IL-17A throughout the infection and required MR1 and IL-12p40 signals from infected antigen-presenting cells to control LVS intracellular growth. In *MR1*^−/−^ mice (lacking MAIT cells), pulmonary LVS infection showed defects in early mucosal cytokine production, timely recruitment of IFN-*γ*–producing CD4^+^ and CD8^+^ T cells to the lungs, and control of pulmonary LVS growth.

MAIT cells promote early differentiation of CCR2-dependent monocytes into monocyte-derived DCs (Mo-DCs) in the lungs after LVS pulmonary infection. Transferring Mo-DCs to MAIT cell-deficient (*MR1*^−/−^) mice corrected their defect in recruiting activated CD4^+^ T cells to the lungs. MAIT cell-dependent GM-CSF production stimulates monocyte differentiation *in vitro*, and GM-CSF production was delayed in *MR1*^−/−^ mice. GM-CSF-deficient mice showed a similar defect in monocyte differentiation to *MR1*^−/−^ mice. These findings demonstrate that MAIT cells drive early pulmonary GM-CSF production, promoting the differentiation of inflammatory monocytes into Mo-DCs (Meierovics and Cowley, 2016).

Jesteadt et al. (2018) found that LVS-infected macrophages produced little IL-18 and low MAIT cell IFN-*γ*. IL-18-deficient macrophages also failed to induce substantial IFN-γ in MAIT cells. However, *in vivo*, IL-18-deficient mice and WT C57BL/6 mice had similar IFN-*γ* levels in MAIT cells during LVS pulmonary infection. This suggests that while IL-18 is crucial for the MAIT cell IFN-γ response *in vitro*, other signals drive MAIT cell IFN-γ production *in vivo*, highlighting the complex cytokine interactions during *Ft* infections.

Also using C57BL/6 mice infected *via* the pulmonary route with LVS as a model, Yu et al. (2020) demonstrated that LVS infection resulted in a large number of MAIT cells in the lungs. These MAIT cells were predominantly chemokine receptor 6 (CXCR6)^+^, although CXCR6 was not required for MAIT cell accumulation in the lungs. CXCR6 was found to contribute to long-term retention of MAIT cells in the airway lumen following resolution of the infection. The authors also found MAIT cells were not recruited from secondary lymphoid organs and largely proliferated *in situ* in the lungs following infection. This work was built upon by Zhao et al. (2021), using LVS. The authors found that during systemic infection of C57BL/6 mice with LVS, MAIT cells expanded in the liver, lungs, kidney, spleen, and peripheral blood. The responding MAIT cells exhibited a polarized transcription factor and cytokine profile characteristic of a Th1-like MAIT-1 phenotype, which was critical in controlling bacterial numbers. Following resolution of the primary infection, the expanded MAIT cells formed stable memory-like MAIT-1 cell populations. This finding of a MAIT memory cell population suggests a possible correlate of protection following vaccination, although repeating the study with a virulent strain would strengthen this association.

#### 3.5.5 Natural killer cells

NK cells are lymphoid cells that are phenotypically and functionally distinct from T cells and have crucial innate immune activity, operating to bridge innate and adaptive immune functions. They secrete several cytokines, including IFN-*γ*, TNF-α/β, CD95/FasL, and TNF-related apoptosis-inducing ligand (TRAIL) (Belizario et al., 2018). NK activity is initiated very soon after infection with *Ft*. Studies by Natrajan et al. (2019) evaluated peripheral blood mononuclear cells (PBMC) from humans vaccinated with either of two sources of LVS (USAMRIID-LVS or DVC-LVS) for a variety of activation signals, including assessment of NK-specific markers (CD56dimCD16-CD69high). These investigators found that NK cell activation increased at Day 1 after immunization, reached peak levels by Day 2, and decreased to pre-vaccination levels by Day 7 post-immunization. There were no differences found in activation between the two vaccine sources. Studies by Lopez et al. (2004) found a dramatic increase in the numbers of cells secreting IFN-γ observed 72 h after i.n. infection of BALB/c and C57BL/6 mice with sublethal (1,000 CFU) or lethal (10,000 CFU) doses of LVS and that the cells primarily responsible for this expression were CD11b^+^ DX5^+^ NK cells. The findings were further confirmed in C57BL/6 mice showing that cells responsible for IFN-γ secretion in the lungs were CD11b^+^ DX5^+^ NK1.1^+^. NK cell depletion studies revealed a decrease in the percentage not only of IFN-γ secreting cells, but also a reduced percentage of T cells secreting IFN-γ. The results indicate that NK cells are the early responders responsible for IFN-γ secretion following *Ft* infection. NK cells may be an important source of IFN-γ early in *Ft* infection.

There appears to be an important interaction between B1a cells and NK/NKT cells as described in studies utilizing WT CBA/J mice and CBA/CaHN-Btk^XID^/J (XID due to defective Bruton’s tyrosine kinase) mice. Mice were infected i.n. with SCHU S4 (Crane et al., 2013). XID mice exhibited an enhanced survival as compared to WT mice; furthermore, although the mean time to death was similar between the two strains, lung and spleen burden with SCHU S4 was less in XID mice as compared with WT controls. This difference was determined to be due to the reduced number of IL-10-producing B1a cells in the XID mice; IL-10 is known to downregulate the production of IL-12, which subsequently reduces the production of IFN-γ by NK and NKT cells. These investigators also found an increase in NK/NKT cells in the spleens and lungs of XID mice as compared with WT, and when the XID animals were treated with anti-Asialo GM1 antibodies (which eliminated NK/NKT cells by more than 98%), the animals were less able to control SCHU S4 infection (Crane et al., 2013).

Schmitt et al. (2013) also evaluated the role of NK cells in controlling *Ft* infection. In their studies, C57BL/6 J mice were NK-depleted using either anti-asialo GM1 or anti-NK1.1 antibodies, and NK cells were augmented by administration of a complex of IL-15 + IL-15Rα. The mice were infected with either LVS or SCHU S4 and cell populations in the lungs were subsequently evaluated over time. Although no significant differences in the frequency or absolute number of T cells or NK cells were observed in LVS-infected mice compared to controls, NK cells and T cells decreased by approximately 90% in the lungs of SCHU S4-infected mice. Also, in LVS-infected mice, IFN-γ was detected at three dpi in the lung and continued to increase until seven dpi. However, IFN-γ levels peaked three dpi with SCHU S4 and declined four dpi. Depletion of NK cells in SCHU S4-infection mice resulted in reduced IFN-γ and granzyme B levels in the lung, although it did not change bacterial burden. Conversely, administration of the IL-15 + IL-15R*α* complex, which is anti-apoptotic, was demonstrated to significantly increase the frequency of NK cells and T cells in infected mice as well as to downregulated inflammatory cytokines including IL-6 and IFN-γ; however, this was not accompanied by any alteration in the progression of disease. The authors concluded from these results that NK cells do not appear to contribute to the control of acute type A *Francisella* infection.

In addition to their ability to secrete immunomodulatory cytokines, NK cells have cytolytic/cytotoxic granules containing perforin, granulysin, and granzymes A and B which they employ in microbial host defense. NK cells have long been studied for their ability to control cancer and viral infections, and it is only more recently that their ability to control bacterial - and especially intracellular bacterial - infection has been investigated. For example, NK cells have been shown to induce programmed cell death in cells hosting intracellular pathogens, using a variety of lytic molecules (Belizario et al., 2018), although there have been relatively few studies evaluating this function relative to *Ft* infection. In one study conducted to evaluate protection against *Ft* induced by LVS using different *Francisella novicida*-based vaccines, WT C57BL/6 and perforin-deficient (*PRF*^−/−^) mice were immunized with the vaccines and then challenged with LVS. Although there were no appreciable differences between the ability of WT and perforin-deficient mice to control a lethal infection with LVS, a differential level of protection was afforded by the two different vaccines. The authors followed up the challenge study with mechanistic evaluation and demonstrated the perforin and ADCC activity was responsible for this difference (Sanapala et al., 2012). Although this is an interesting observation, it is unclear whether perforin plays a major role in NK cell control of *Ft*. To date, there does not appear to be any significant ongoing research in this area, and it seems unlikely that this would be a promising line of investigation for further insights into *Ft* pathophysiology.

#### 3.5.6 Polymorphonuclear cells (neutrophils)

Neutrophils are crucial for controlling primary *Ft* infections and resisting reinfection (Sjostedt et al., 1994). While lung macrophages are the principal targets upon respiratory exposure, both LVS and SCHU S4 can infect and replicate within neutrophils to a similar extent (Hall et al., 2008). Early in the infection, neutrophils are heavily recruited, but their bactericidal mechanisms are inhibited. Over time, immature myeloid cells and myeloid-derived suppressor cells dominate, eventually dying in the lungs and causing a necrotizing cascade that damages the lungs and leads to host death. This pattern is seen with both SCHU S4 and LVS, though LVS requires higher doses to replicate SCHU S4’s effects (Periasamy et al., 2016).

In addition to *Ft*’s ability to subvert inflammation and thus escape destruction by neutrophils, *Ft* contributes to overall pathology by prolonging survival of neutrophils. Kinkead et al. (2022) employed *in vitro* cultures of purified human neutrophils infected with LVS to monitor apoptosis in the presence of various inhibitors. The study showed that both ERK2 and p38α were activated in *Ft*-infected neutrophils, but only p38α MAPK was required for delayed apoptosis, revealing another mechanism whereby *Ft* enhances its survival in the host while producing significant pathology. Another mechanism of neutrophil pathology was demonstrated by Pulavendran et al. (2020), who showed that BALB/c mice infected with LVS exhibited a large number of neutrophil extracellular traps (NETs), which harbored large numbers of *Ft*, although without producing any bactericidal activity. While there were changes in ERK2 and p38α in *Ft*-infected neutrophils, we founded no evidence that this is linked with a memory or recall immune response.

#### 3.5.7 Mast cells

Mast cells have historically been associated with type 1 hypersensitivity; however, their ability to produce a variety of soluble bioactive molecules (histamine, leukotrienes, prostaglandins and a variety of cytokines such as TNF-α and IL-4), their ability to phagocytose microorganisms, their bactericidal capability, and their geographic location in the lungs suggests a potential role in controlling bacterial infections including *Ft*. To evaluate whether mast cells are important in *Ft* infections, Ketavarapu et al. (2008) infected C57BL/6 and BALB/c mice i.n. with LVS and subsequently evaluated the lungs and cervical (draining) lymph nodes for inflammatory cells (macrophages, neutrophils, and mast cells) by flow cytometry. The investigators found that mast cells accumulated within the lungs earlier than other inflammatory cell populations. Employing *in vitro* culture systems with bone marrow-derived cells infected with LVS revealed only limited replication of LVS within mast cells as compared with a robust replication within macrophages. However, when the bone marrow-derived cells were co-cultured and infected with LVS, there was a significant reduction in LVS replication within macrophages. Follow-up *in vitro* studies demonstrated that IL-4 production by mast cells was a contact-independent mechanism controlling LVS replication in macrophages. Rodriguez et al. (2011) showed this activity to be mediated by increased ATP production and phagosomal acidification. Subsequent studies by this group using *in vitro* culture of mast cells with either LVS or SCHU S4 demonstrated that mast cells require TLR2 for effective bacterial killing, regulation of the hydrolytic enzyme cathepsin L, and for coordination and trafficking of MHC-II and lysosomal associated membrane protein 2 (LAMP2) (Rodriguez et al., 2012). Hunter et al. (2012) have shown that mucosal mast cells are phenotypically like bone marrow-derived mast cells (as assessed by expression of FcεRI, c-Kit, and MHC-I) and can limit intramacrophage *Ft* replication *via* elucidation of IL-4. This is important in that it demonstrates a tissue-relevant function for these cells.

#### 3.5.8 Innate lymphoid cells

Innate lymphoid cells (ILCs) are tissue-resident cells, divided into three groups, ILC1, ILC2, and ILC3. ILCs are defined by high expression of CD90.2 and CD127. Following i.n. infection of C57BL/6 mice with LVS, there was a decrease in ILC2 and bacterial burdens. Enhancing ILC2 numbers with IL-33 resulted in more LVS in lung, liver, and spleen. IFN-*γ* contributed to the reduction of ILC2s and the ILC2 enhancement of bacterial burden could be reduced by blockade of IL-5 (Dow et al., 2023). A more comprehensive understanding of these ILCs and how they interact during tularemia is needed.

## 4 Discussion

Historically, research on immunity to *Ft* has centered on B cell antibody production, T cell functions (particularly relating to cytokine roles), and the contributions of macrophages and antigen-presenting cells in processing antigens and coordinating cellular and humoral responses (Rhinehart-Jones et al., 1994; Koskela and Herva, 1982; Cowley and Elkins, 2011). Whereas these basic components have been studied extensively by many investigators, the full host immune system interaction with *Ft* is far from established (Bosio, 2011; Jones et al., 2011; Bahuaud et al., 2021). In the present review, we have endeavored to summarize more recently (approximately the past 10 years) studied mechanisms including *Ft*’s ability to modulate host immunity, the role of the inflammasome in successful (and unsuccessful) immune responses, the panoply of cytokines that modulate the immune and inflammatory responses, and a variety of both innate and adaptive cellular effectors including γδ T cells, NKT cells, MAIT cells, neutrophils and mast cells. Despite this wealth of added information, an overarching understanding of this complex interactions between host and pathogen remains elusive, for numerous reasons summarized below.

### 4.1 *Ft* is inherently immunomodulatory

An underappreciated feature of *Ft* infection is the organism’s ability to deploy a variety of molecular mechanisms, enabling it to evade and survive the host’s immune response. One surprising mechanism is the post-transcriptional regulation of SHIP-1 via miR-155 while *Ft* simultaneously suppresses MyD88 activity, selectively disinhibiting one of the two pathways downstream of TLR2 (Bandyopadhyay et al., 2014; Cremer et al., 2011). This selective activation and suppression of specific immunological pathways, even for the same receptor, highlight *Ft*’s sophisticated immune evasion strategies. Inflammasome pathways interact extensively with TLR2 and TLR4, which are intricately linked with *Ft* pathophysiology. The pathways for these receptors produce IFN-*α* and -*β*, activating mechanisms for programmed cell death by necrosis, apoptosis, and pyroptosis. *Ft* can prevent the activation of pyroptosis receptors AIM2 and NLRP3, which interact with caspase-1 and cytokines like IL-18 to trigger cell death. Redundant regulation mechanisms of these pathways by negative feedback loops and external factors such as c-Abl and IFN-β also suggest that further research into these pathways may be beneficial, given that inflammasome signaling can trigger multiple cell death pathways at once. *Ft* does not trigger responses from these receptors, causing deficiencies, particularly in macrophage activation and subsequent signaling that trigger the proper response to a pathogen (Juruj et al., 2013; Corrales et al., 2016; McCracken et al., 2016). Finally, *Ft* also suppresses ROS, which are crucial for destroying intracellular pathogens. *Ft* can evade ROS destruction through mechanisms like SodB and OxyR activity and by preventing nitrite production in host cells. These strategies neutralize or inhibit host ROS production, creating a favorable environment for *Ft*’s survival and replication (Ma et al., 2016; Honn et al., 2017; Rabadi et al., 2016; Gillette et al., 2014). Although many of the studies of *Ft*’s immunomodulatory activities are descriptive, we believe that a mechanistic understanding of *Ft*’s ability to down-modulate immunity is important to consider as a component of MCM development.

### 4.2 Animal model differences clearly lead to dissimilar/conflicting results

While mammalian immune systems share many essential features, it is axiomatic that humans and rodents are dissimilar in many ways. These fundamental differences between human and murine immune responses are a key factor that undermines conclusions based solely on studies of murine models (Nauseef, 2001; Zschaler et al., 2014). This impacts responses to *Ft* and the ability to translate all mouse data to the human clinical outcomes.

The choice of the most appropriate animal models is crucial when developing MCM against *Ft* that require use of the Animal Rule (Roberts et al., 2018). Our review of the literature revealed that an overwhelming majority of studies have used mice as the animal model (Figure 1); this is unsurprising since mice are a traditional model for immunological studies, their immune systems are well characterized, and they are readily available and affordable for performing *in vivo* studies. However, there are numerous and often significant differences between the immune response of these species (Mestas and Hughes, 2004), making a direct comparison imprecise at best. Even within mice, there are strain differences. The studies reviewed here showed that BALB/c mice and C57BL/6 mice to be the most common strains used, although it is unclear in many cases why one strain was selected over another. The known BALB/c and C57BL/6 strain differences predispose to certain cytokine profiles, for example, macrophages from C57BL/6 mice produce higher levels of TNF and IL-12 compared to macrophages from BALB/c mice (Watanabe et al., 2004) in an *in vitro Ft* exposure culture.

Despite the concerns associated with using primates, greater emphasis should be placed on using this model. Cynomolgus macaques appear to more closely approximate human immune responses to *Ft* than rodents (Glynn et al., 2015; Guina et al., 2018; Frick et al., 2021). In addition, considerable progress has been made with experimental systems including transgenic animals (Oh et al., 2018), cell co-culture systems (De Pascalis et al., 2018; David et al., 2014; Eneslatt et al., 2018; Koppen et al., 2023) and *ex vivo* organ culture (Rennert et al., 2016). These systems will require further refinement but show promise in understanding *Ft*’s immune response and may lead to assays for immune correlates of protection and provide valuable bridges between human and nonhuman data. A combination of nonhuman primate data and *ex vivo* human data, supplemented by well-controlled rodent data, would be an appropriate pathway for Animal Rule compliance.

### 4.3 Inconsistency in infecting/challenge Ft strain may be problematic

A preponderance of the literature reveals the use of the LVS strain as a challenge model, although for development of MCM this is not an appropriate choice. Although LVS and SCHU S4 exhibit up to 99.8% homology in their genomes (Kudryavtseva TaM, 2022), the fact remains that these strains often produce differing results in both *in vitro* and *in vivo* models. Notwithstanding technical challenges associated with high-risk infectious agents, to better support MCM development, more emphasis should be placed on strategies to evaluate the immune response to virulent strains of *Ft* such as SCHU S4.

### 4.4 Immunization and challenge routes are important

Inconsistency in route of vaccination and challenge routes may be problematic since disease type and outcome vary with exposure route (Degabriel et al., 2023). As reviewed by Nicol et al. (Nicol et al., 2021), data generated in rodents, rabbits, NHP, and humans reveal differences in immune response following i.n. and i.d. infection. For MCM development, it will be important to standardize both immunization and challenge routes, preferably using aerosol exposure.

### 4.5 Immunity to *Ft* depends on far more than T and B cells

Although humans and other animals are known to mount a humoral immune response following exposure to *Ft*, the historical data remain inconclusive as to the protective value of this response. Likewise with cellular immunity, multiple investigators have developed extensive cytokine panels to understand the role of these mediators (Lindgren et al., 2023), correlations remain elusive, especially between humans and other species. As demonstrated by the present review, effective defense against *Ft* is likely to require multiple innate and adaptive responses working in concert. For MCM development, what is needed are approaches that incorporate multiple effectors.

*Ft*’s ability to evade cellular and molecular defenses, combined with its potential for weaponization, underscores the need for additional interventions. Although current antibiotics are effective, the risk of *Ft* developing antibiotic resistance, either naturally or through genetic engineering, highlights the need for prophylactic solutions as a component of a layered defense strategy. Despite decades of effort, no tularemia prophylactic, vaccine or otherwise has been approved by the FDA. Based on the Animal Rule guidance on data linking the effective dose to human patients and animal models, we recognize immune correlates of protective immunity should be examined across human and animal species. According to Plotkin (Plotkin, 2020; Plotkin, 2022), a correlate of protection is “an immune function that correlates with and may be biologically responsible for vaccine-induced efficacy.” What is needed are concerted efforts to specifically evaluate the multitude of immune effectors that have been identified as contributing to immunity to *Ft* so that correlates of protection can be incorporated into effective MCM for tularemia.
